# Supplementary material for: Stool metabolome-microbiota evaluation among children and adolescents with obesity, overweight, and normal-weight using 1H NMR and 16S rRNA gene profiling
Source: PLoS One. 2021 Mar 25;16(3):e0247378. doi: 10.1371/journal.pone.0247378 (PMC7993802; doi:10.1371/journal.pone.0247378)
Supplement: Attachment — Submitted filename: Response_to_Reviewers.doc [file pone.0247378.s002.doc]

November 26, 2020

Dear Editor and Reviewers,

We would like to extend a sincere thank you for your valuable feedback. We have carefully addressed all your comments, and our revised and improved manuscript reflects this.

The main changes concentrated on providing more details regarding the study group’s characteristics and clinical/demographic profile, as well as further detail on the diet consumed by the participants. These changes are mainly reflected in the methods subsection “Study participants.” Per the reviewers’ feedback, Fig 3 was removed and Fig 4 (now Fig 3) was changed to a heatmap with the focus mainly being the inter-individual variability of the participants at the phylum level. Former Fig 5 is now Fig 4, and a new heatmap showing all metabolite-genera correlations has been added as Fig 5. These, as well as further edits and additions in the text, are mentioned in detail in the response to the reviewers that follows this letter. Seven additional citations were added per these changes.

Further details to note are the addition of another co-author, Lucie Vodolánová, for her investigative contribution about the gut bacteria. This change also altered the acknowledgments section as well as the online description of authors’ contributions. Regarding the data availability, we will use, as originally planned, to place it in Mendeley Data under the following DOI link: http://dx.doi.org/10.17632/cwj76cbvc9.1. The items to be included there are: 1) S1 Table 1 (as referenced in the manuscript); 2) Unrarefied source data for the 16S rRNA gene sequencing analysis; 3) Metabolite concentrations in mg/g; and 4) the ^1^H NMR spectra.

We believe that our article adds valuable information to the growing research on obesity during childhood and adolescence, especially regarding potential metabolic signatures of the condition. Thank you for your time and consideration.

Sincerely,

Jaroslav Havlík, Ph.D.

Senior lecturer in Agricultural Chemistry

Department of Food Science, Czech University of Life Sciences Prague

e-mail: havlik@af.czu.cz tel.

+420 777 558 468

**Response to reviewers.**

**Reviewer 1:**

**Comment:**

1. Given the focus on obesity, metabolites, and diet, the description of diet in this study is lacking on multiple levels.

a. The statement that diet was controlled in this study by simply balancing macronutrients (fats, carbohydrates, and protein) is a misleading overstatement. Given these constraints, one subject could have eaten simple carbohydrates (e.g., highly processed white bread) and another subject could have eaten an equivalent caloric percentage of carbohydrates of completely complex carbohydrates (e.g., fruits, vegetables), and these would be considered the same; however, these diets would lead to extremely different microbiota and metabolomes.

**Response:**

We agree with your comment and have now provided more detailed information about the diet that the participants followed. The composition of the meals among the participants was similar (similar ingredients, similar dishes); for example, the carbohydrate portion of each meal was of the same food item, differing only in the portion size based on the subject’s age, gender, and weight. This information has now been more explicitly stated in the methods subsection “Study participants.” Additionally, new information includes the macronutrient percentage intake per each of the three groups (normal, overweight, obese) one and two days prior to sampling (Table 1), as well as per each of the participants (S1 Table 1). This has now been more clearly stated (lines 127-133).

**Comment:**

b. Additionally, there is no description of calorie controls beyond the statement that “the children with obese/overweight on a caloric restriction”. Given that half of the metabolites identified (arabinose, galactose) are found in food and do not require transformation by microbial metabolism, a highly plausible explanation is that subjects in the OW and OB groups consumed more food that resulted in the higher amounts of arabinose and galactose in the stool. If diet is to be considered in this manuscript, there must be a complete explanation and analysis of diet as a co-factor.

**Response:**

We have now explicitly stated that those with overweight/obesity were in a 30 % caloric restriction (lines 129-130). Additionally, daily kilocalorie intake per each of the three groups (normal, overweight, obese) one and two days prior to sampling (Table 1), as well as per each of the participants (S1 table 1), has now been included. The diet has been more clearly presented through these tables and in the methods section (lines 127-133). We believe that the new provided information more accurately reflects the consideration of diet as a co-factor and the potential bias it may introduce in the interpretation.

**Comment:**

The control group (normal weight) is a group that was receiving treatment of respiratory or locomotive conditions. Individuals with underlying and potentially confounding conditions should not be used as controls.

**Response:**

We agree that the control group (normal-weight youth) may not be an ideal control group; however, given the selection criteria, we still believe that they represent a valuable comparison group. The controls with a respiratory disease were in remission of their condition, and those with an orthopedic (locomotive) diagnoses were healthy enough to take part in the program’s physical activity regime. In addition, the recruitment criterion was no antibiotics in the past three months prior to program participation, none were taking medications, and all were physically able to take part in a physical activity program. Furthermore, except for portion size, they all received a similar diet (same ingredients, same dishes). As a result of the selection criteria, a similar physical activity regime, a homogenous diet among the participants, and the same ethnic and geographic background of the participants, the effect of cofounding variables was minimized. Consequently, we believe that the observations from our study are relevant in contrasting the metabolomic and gut microbiota differences among the three groups (normal-weight, overweight, obese) in children/adolescents. The section describing this (lines 113-135) has been updated to provide these and further details.

**Comment:**

A complete description, or reference, is needed to describe the mock community used as a reference.

**Response:**

Further details have been provided to describe all the participants, including the controls. (lines 113-135, Table 1, S1 Table 1)

**Comment:**

The relationship between gut microbiome and obesity has been examined extensively in many studies with large sample sizes. For a summary see:

Sze, M.A. and Schloss, P.D., 2016. Looking for a signal in the noise: revisiting obesity and the microbiome. MBio, 7(4), pp.e01018-16. Additional validation of these trends is not needed.

The same holds for the SCFA connection, for example

Schwiertz, A., Taras, D., Schäfer, K., Beijer, S., Bos, N.A., Donus, C. and Hardt, P.D., 2010. Microbiota and SCFA in lean and overweight healthy subjects. Obesity, 18(1), pp.190-195.

Kim, K. N., Yao, Y., & Ju, S. Y. (2019). Short Chain Fatty Acids and Fecal Microbiota Abundance in Humans with Obesity: A Systematic Review and Meta-Analysis. Nutrients, 11(10), 2512. https://doi.org/10.3390/nu11102512

The authors should consider refocusing the analyses on the age of the subjects (most studies have been conducted in adults) and/or additional types of analyses that have not been previously conducted.

**Response:**

Although it has been examined, the findings are still far from being established. There is ample room for expansion and provide a more complete and precise picture of the metabolomic-gut bacteria changes involved.

In regard to focusing the analyses on the age of the subjects, that is actually one of our aims since, as you mentioned, most students have been focused on adults. To express this aim more explicitly, we have revised the text (lines 65, 75-77, 87-91). This included the addition of an additional citation (Radjabzadeh D, Boer CG, Beth SA, van der Wal P, Kiefte-De Jong JC, Jansen MAE, et al. Diversity, compositional and functional differences between gut microbiota of children and adults. Sci Rep. 2020;10: 1–13. doi:10.1038/s41598-020-57734-z).

**Comment:**

Speculations regarding mechanisms (i.e., increased energy harvest) are lacking support and should be revised.

**Response:**

We have elaborated the Discussion section to provide more support for the increased energy harvest mechanism in lines 374-376 as well as other details throughout this section. We do attempt to make it clear in our writing that this is still a proposed mechanism. Several citations (some previously in the manuscript and some new additions) support this mechanism. (Turnbaugh P. J., Ley R. E., Mahowald M. A., Magrini V., Mardis E. R. GJI. An obesity-associated gut microbiome with increased capacity for energy harvest. Nature. 2006;444: 1027–1022. doi:10.1007/s11837-013-0766-1), (Goffredo M, Mass K, Parks EJ, Wagner DA, McClure EA, Graf J, et al. Role of gut microbiota and short chain fatty acids in modulating energy harvest and fat partitioning in youth. J Clin Endocrinol Metab. 2016;101: 4367–4376. doi:10.1210/jc.2016-1797), (Oliphant K, Allen-Vercoe E. Macronutrient metabolism by the human gut microbiome: Major fermentation by-products and their impact on host health. Microbiome. 2019;7: 1–15. doi:10.1186/s40168-019-0704-8), (Morrison DJ, Preston T. Formation of short chain fatty acids by the gut microbiota and their impact on human metabolism. Gut Microbes. 2016;7: 189–200. doi:10.1080/19490976.2015.1134082), (Riva A, Borgo F, Lassandro C, Verduci E, Morace G, Borghi E, et al. Pediatric obesity is associated with an altered gut microbiota and discordant shifts in Firmicutes populations. Environ Microbiol. 2017;19: 95–105. doi:10.1111/1462-2920.13463), (Schwiertz A, Taras D, Schäfer K, Beijer S, Bos NA, Donus C, et al. Microbiota and SCFA in Lean and Overweight Healthy Subjects. Obesity. 2010;18: 190–195. doi:10.1038/oby.2009.167), (Payne AN, Chassard C, Zimmermann M, Müller P, Stinca S, Lacroix C. The metabolic activity of gut microbiota in obese children is increased compared with normal-weight children and exhibits more exhaustive substrate utilization. Nutr Diabetes. 2011;1: e12–e12. doi:10.1038/nutd.2011.8).

**Comment:**

Please consider changing the type of figure used to display taxonomic abundance (Figure 4). The pie charts currently used are an ineffective and inaccurate method of representing the data, as they do not include any display of variance. Consider using a heatmap to show intersubject variability. Additionally, the Firmicutes/Bacteroidetes appear to be incorrect based on the data presented in this figure (N – 50.4/34.1 = 1.48, not 2.2; OW – 57.9/28.7 = 2.02, not 3.4; OB – 59.3/26.2 = 2.26, not 5.7).

**Response:**

We followed your suggestion and have replaced this figure with that of a heat map to better display inter-individual variability. Since there was no significant difference among the three groups, the percentages from the previous figure were excluded in this figure. The previous Firmicutes/Bacteroidetes discrepancy was due to a calculation that included the outliers that had been excluded in the previous figure.

**Comment:**

Tukey’s HSD provides weak control of Type I error. Please consider using false discovery rate to correct for multiple comparisons in this type of exploratory study.

**Response:**

We have considered this and did notice that our significant metabolites were significant at a false discovery rate of 0.56, which means that we would expect 44 % of the identified metabolites to be significant, except that it is hard to ascertain which ones. Since it is an exploratory study, we would like to keep our current methodology as to not potentially discard valuable metabolites. Other researchers may find this important for generating further hypotheses.

**Reviewer 2:**

**Comment:**

I would like to see a more sophisticated analysis to find associations between microbes and metabolites. The authors only examined microbes and metabolites that were significantly different across BMI groups for positive Spearman correlations (page 12, line 313). An analysis that includes all metabolites and bacteria could reveal other patterns that differ across BMI groups; examples include correlations (seen in Shankar, et al). The sparse partial least squares regression (seen in Cribbs, et al)

**Response:**

We believe that you raise a very important point, and we also agree that an analysis that includes all metabolites and genera would be very valuable to report. We consulted both of the examples you provided, and we proceeded with a Spearman correlation analysis similar to what we had initially done; however, we now included all metabolites and genera detected and have displayed these correlations in the form of a heatmap (Fig 5) in the results subsection “Correlation of metabolomic and 16S rDNA analyses.” Consequently, we have also added some material both in the results and discussion sections.

**Comment:**

You refer to the microbiome but only examined bacteria. A discussion that includes other microbes, or the caveat that only bacteria were examined, would widen the audience and appeal of the study.

**Response:**

We have edited our language throughout the paper to reflect that only bacteria were examined. We still kept the same title, but throughout the text we believe that it is now clearer that we specifically examined bacteria. We agree that the appeal would be widened by including microbiota outside of bacteria; however, our 16s analysis results are better suited to report and focus only on bacteria for this study.

**Comment:**

Lines 77-81: The sentence that starts “For example” is difficult to read and should be simplified to avoid having so many lists in one sentence.

**Response:**

A slight modification of the sentence was made (now line 83-85)

**Comment:**

The correlations between metabolites (lines 175-178, 276-284, and fig 3) doesn’t seem to add anything and I would suggest removing it.

**Response:**

We agree with this suggestion and have removed the figure. We are now reporting the correlation coefficients from the figure in written form in lines 311-316.

**Comment:**

Line 211 - When you removed cyanobacteria, were these all cyanobacteria or only those that could not be classified further (potentially indicating non-bacterial origins)? If the latter, please specify.

**Response:**

We removed all identifiable cyanobacteria signal. Its overall share on the total bacteriome was negligible, only 0.077 % reads. It originated from 5 samples, with the largest signal being 2.2% per sample, and second largest being 0.5 % per sample. These cyanobacteria were classifiable only to the level of order, but all belonged to Gastranaerophilales, an order that - rather surprisingly - consistently lacks both photosynthetic, and aerobic pathways [Rochelle M Soo et al, Science 2017, vol 355, issue 6332, pp. 1436-1440]. The lack of aerobic metabolism makes it probable that the finding is not incidental: these cyanobacteria may indeed be present in the gastrointestinal tract, as the order name suggests and as has been documented by Soo et al in an earlier submission to the Sequencing Reads Archives (https://www.ncbi.nlm.nih.gov/bioproject/PRJNA348149). However, we think it is prudent to delete this negligible portion of reads: (a) the power to detect an association with the outcome is close to zero due to the low abundance of the bacteria, (b) it would confound readers, as cyanobacterium is mostly thought of as a plant predecessor, an organism that can photosynthesize; thus it is often automatically removed like we did.

**Comment:**

Line 237 - There’s an extra comma between “or greater” and “a variance >10%”.

**Response:**

It has been addressed (now line 271).

**Comment:**

Line 340 - This is the second sentence in a row to start with “Also”. Consider using “Finally”, another transition word, or nothing at all.

**Response:**

The transition word was changed to “In addition” (now line 413).

**Comment:**

Line 345 - I would suggest you move the reference to Fig 3 to after “with each other” which is what is displayed in the figure - if you decide to keep it, see above.

**Response:**

We decided to remove the figure per your previous comment about it.

**Comment:**

Line 370 - Please specify that it is BMI z-score

**Response:**

We have now specified that it is BMI z-score (now line 443). Similar specifications were made throughout the manuscript.

**Additional requirements:**

**Comment:**

Please ensure that your manuscript meets PLOS ONE's style requirements, including those for file naming. The PLOS ONE style templates can be found at

https://journals.plos.org/plosone/s/file?id=wjVg/PLOSOne_formatting_sample_main_body.pdf and

https://journals.plos.org/plosone/s/file?id=ba62/PLOSOne_formatting_sample_title_authors_affiliations.pdf

**Response:**

We have followed the style requirements per the comment, and our revised manuscript reflects this.

**Comment:**

We note that you have stated that you will provide repository information for your data at acceptance. Should your manuscript be accepted for publication, we will hold it until you provide the relevant accession numbers or DOIs necessary to access your data. If you wish to make changes to your Data Availability statement, please describe these changes in your cover letter and we will update your Data Availability statement to reflect the information you provide.

**Response:**

The data will be provided via Mendeley Data as we had originally planned under the following DOI link: http://dx.doi.org/10.17632/cwj76cbvc9.1. The items to be included there are: 1) S1 Table 1 (as referenced in the manuscript) containing the individual participant’s gender, age, BMI, BMI z-score, as well as the kilocalorie and macronutrient daily percentage composition one and two days prior to sampling; 2) unrarefied source data for the 16S rRNA gene sequencing analysis; 3) Metabolite concentrations in mg/g derived from Chenomx NMR Suite version 7.5 for each of the 52 study participants; and 4) the ^1^H NMR spectra for the 52 study participants.

**Comment:**

In your Methods section, please provide additional information about the participant recruitment method and the demographic details of your participants. Please ensure you have provided sufficient details to replicate the analyses such as: a) the recruitment date range (month and year), b) a description of any inclusion/exclusion criteria that were applied to participant recruitment, c) a table of relevant demographic details, c) a description of how participants were recruited.

**Response:**

This information has now been more explicitly stated in the methods subsection “Study participants.”

**Comment:**

We suggest you thoroughly copyedit your manuscript for language usage, spelling, and grammar. If you do not know anyone who can help you do this, you may wish to consider employing a professional scientific editing service.

**Response:**

We have copyedited our manuscript. All revisions can be followed via Track Changes.

**Comment:**

Please provide a sample size and power calculation in the Methods, or discuss the reasons for not performing one before study initiation.

**Response:**

Given that this is an exploratory pilot study, we did not have a clear hypothesis about which metrics would be expected to be different among the study groups, thus an accurate power calculation did not seem suitable nor feasible. Also, the study was limited in time and funding, thus we were limited on the cohort size and recruitment time possibilities.
