# Supplementary material for: Stool metabolome-microbiota evaluation among children and adolescents with obesity, overweight, and normal-weight using 1H NMR and 16S rRNA gene profiling
Source: PLoS One. 2021 Mar 25;16(3):e0247378. doi: 10.1371/journal.pone.0247378 (PMC7993802; doi:10.1371/journal.pone.0247378)
Supplement: Attachment — Submitted filename: Response_to_Reviewers.doc [file pone.0247378.s003.doc]

January 7, 2021

Dear Editor and Reviewers,

Once again, we thank you for helping us improve the presentation of our findings. We have responded to the reviewers’ comments in the pages following this letter. In addition to edits within the text, we have also added one new figure (Fig 4) and a new reference.

We look forward to your reply and are very appreciative for your time and consideration.

Sincerely,

Jaroslav Havlík, Ph.D.

Senior lecturer in Agricultural Chemistry

Department of Food Science, Czech University of Life Sciences Prague

e-mail: havlik@af.czu.cz tel.

+420 777 558 468

**Response to reviewers.**

**Reviewer 1:**

**Comment:**

I appreciate the author’s providing more information about diet. I still have some additional questions/concerns. Was this an outpatient or inpatient study? If inpatient I assume that diet was controlled by the staff (although explicit information would be helpful). However, if this was an outpatient study, how was dietary management implemented? Were meals provided by the study, or were meals prepared by subjects/subject’s families? Additionally, compliance with dietary guidance is a well-established issue in outpatient studies. Was this controlled at all? Finally, how long were subjects on the controlled diet prior to sampling? This would be important to determine whether the observed changes were a response to stimuli (diet) or a new steady-state composition/function of the microbiome and metabolites.

**Response:**

We have elaborated on this information in the subsection Study Participants. It was an inpatient study (lines 111-112, 127), the diet was planned by a clinical dietitian, and the food prepared by the cafeteria kitchen of the Olivova Children’s Medical Institution and (lines 131-133). For further clarity, we added more details about the diet in lines 131-138 and added a statement regarding compliance (leftover food and/or additional intake of other items outside the provided meals could not be accounted for) in lines 139-140. The participants were not all sampled at the same time and sampling took place throughout the study period with the earliest collections taking place after at least one week of habituation to the prescribed diet (lines 142-144). Also, as noted in lines 137-138, the meals were based on recipes and foods typically eaten in a standard Czech school and home diet, thus, except for portion control, the meals did not represent an adjustment for most participants.

**Comment:**

I appreciate the additional information regarding control subjects, thank you! It might be interesting to compare reference data sets (e.g., HMP, other pediatric studies) to determine whether the controls in this study are similar to others, but that does not need to be a requirement for publication.

**Response:**

We did not add this to the manuscript, but we did take a look at some other control groups in studies that also divided youth into normal, overweight, and obese categories. The following table shows how the normal-weigh controls in our study compared with theirs in regard to sample size, gender, age, and BMI.

|  | **Our study** | **Study A** [1] | **Study B** [2] | **Study C** [3] |
| --- | --- | --- | --- | --- |
| # Controls / *n* | 16 / 52 | 15 / 84 | 37 / 102 | 62 / 126 |
| Gender | M = 7, F = 9 | M = 7, F = 8 | M = 18, F = 19 | 31 /31 |
| Age (years) | 11.06 (± 2.46) | 15.42 (± 3.01) | 13.0 (± 2.70) | 6.8 (± 2.4) |
| BMI (kg/m^2^) | 18.05 (± 2.45) | 21.43 (± 2.38) | 18.0 (± 1.1) | 15.2 (± 1.6) |

1.	Goffredo, M.; Mass, K.; Parks, E.J.; Wagner, D.A.; McClure, E.A.; Graf, J.; Savoye, M.; Pierpont, B.; Cline, G.; Santoro, N. Role of gut microbiota and short chain fatty acids in modulating energy harvest and fat partitioning in youth. *J. Clin. Endocrinol. Metab.* **2016**, *101*, 4367–4376, doi:10.1210/jc.2016-1797.

2.	Bervoets, L.; Massa, G.; Guedens, W.; Reekmans, G.; Noben, J.P.; Adriaensens, P. Identification of metabolic phenotypes in childhood obesity by H NMR metabolomics of blood plasma. *Futur. Sci. OA* **2018**, *4*, doi:10.4155/fsoa-2017-0146.

3.	Gao, X.; Jia, R.; Xie, L.; Kuang, L.; Feng, L.; Wan, C. Obesity in school-aged children and its correlation with Gut E.coli and Bifidobacteria: A case-control study. *BMC Pediatr.* **2015**, *15*, doi:10.1186/s12887-015-0384-x.

**Comment:**

I appreciate the additional information on participants, but the comment was in regard to the mock community used for sequencing controls.

**Response:**

We have now added the detailed composition of the mock community (lines 238-244).

**Comment:**

I appreciate the expanded discussion, thank you! I would recommend revising the wording of the statement “Despite consuming less kcal, the OW and OB groups still produced significantly more butyrate, which has been identified as the main energy supplier for colonic epithelial cells [8].” The measurement in this case is of butyrate in feces – which is butyrate produced by the microbiota but not consumed by the colonic epithelium.

**Response:**

We have revised the wording of this statement to be clear that there was a higher content of fecal butyrate, but not necessarily that more butyrate was produced (lines 398-401).

**Comment:**

This new figure conveys much additional information but is only at the phyla level. It would be helpful to include a genus level figure in addition to (not in replace of) the phyla level results. Additionally, the unsupervised clustering really shows the lack of segregation of microbial communities by group. I would suggest adding to the discussion the notion that while there are minimal differences in microbial community composition between groups, the metabolite data suggests that there may be differences in the metabolic activity of these microbes.

**Response:**

To depict data from another angle, we have now added a stacked bar charts figure at the genus level (Fig 4) showing the 20 most abundant taxa (by relative abundance), as well as the two genera which are significant (*Escherichia* and *Tyzzerella* subgroup 3). To keep the figure more readable and due to the very low relative abundances of most taxa, not all 83 identified genera were shown in the figure. We also added on the notion that gut microbiota functional activity, rather than composition, may have played a part in our observations (lines 479-487).

**Comment:**

Thank you for the response. I think that this is fine but should be mentioned in the manuscript. Perhaps a more appropriate way to address this is to state that, while the significance of the metabolites does not survive correction using FDR, there are trends of interest given the sample size in this study. I agree that this data is an important and useful addition to the field!

**Response:**

As suggested, we have now mentioned and addressed FDR in the manuscript (lines 220, 315-319).

**Reviewer 2:**

**Comment:**

Lines 313 and 314, replace p<0.001 with exact p-values, if possible.

**Response:**

We have not made this change due to the p-values being quite small (p = 2.220E-16 for acetate with butyrate, p = 3.363E-06 for arabinose with galactose), and to keep consistency with the number of decimal places for the other reported p values.

**Comment:**

Line 398, consider replacing “authors” with “instigators”, only because I was immediately looking for authors of another study.

**Response:**

Edited as suggested (line 415).

**Comment:**

Line 422 unnecessarily uses “directly” twice; I would suggest removing the second usage.

**Response:**

Edited as suggested (line 439).

**Comment:**

For showing taxonomic abundance (Figure 3), I prefer stacked barcharts, but this is clearly a personal preference and given that the other reviewer specifically asked for a heatmap it works. Either is preferable to a pie chart.

**Response:**

Bar charts are a very good way to represent this information, and we have now added a stacked bar chart (Fig 4) based on genera % relative abundance to provide another angle to look at the taxonomic data.
